# Autosomal recessive RYR2 genetic mutation presenting as atrioventricular block and polymorphic ventricular tachycardia in a young female

**DOI:** 10.1016/j.hrcr.2024.11.013

**Published:** 2024-11-21

**Authors:** Sanjai Pattu Valappil, Narayanan Namboodiri, Shaima Hafeez, Abhinav B. Anand

**Affiliations:** 1 SRM Institute of medical sciences, No 1, Vadapalani, Chennai, India; 2 Sree Chitra Tirunal Institute for Medical Sciences & Technology, Trivandrum, Thiruvananthapuram, Kerala, India; 3 SRM Institute of medical sciences, Vadapalani, Chennai, India; 4 Department of Cardiology, Lokmanya Tilak Municipal General Hospital and Medical College, Sion Hospital, Mumbai, India

**Keywords:** Polymorphic ventricular tachycardia, CPVT, RYR2 mutation, Autosomal recessive, Syncope

**Key teaching points**

• The clinical spectrum of catecholaminergic polymorphic ventricular tachycardia varies and includes progressive AV block, SA node dysfunction, atrial fibrillation, arrhythmogenic right ventricular dysplasia features, and polymorphic ventricular tachycardia.
• Autosomal recessive catecholaminergic polymorphic ventricular tachycardia can present without a family history of sudden cardiac death.
• In a young patient with symptomatic AV conduction disease and absence of inflammatory or structural heart disease, genetic disorders should be strongly considered.

## Introduction

A young woman in her thirties, born out of consanguineous marriage, with no family history of sudden cardiac death, initially presented with symptomatic high-grade atrioventricular (AV) block, for which she underwent a dual-chamber left bundle branch area pacemaker implantation. She subsequently developed recurrent episodes of polymorphic ventricular tachycardia (PVT) and was found to have an autosomal recessive mutation for catecholaminergic polymorphic ventricular tachycardia (CPVT).

## Case Presentation

**Figure 1:**
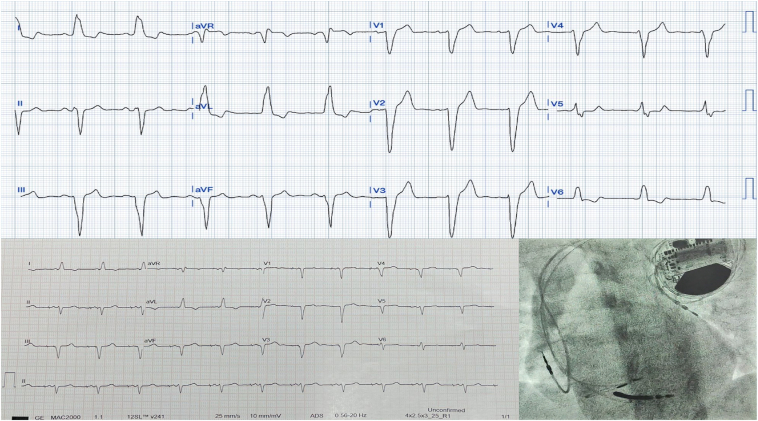
Baseline ECG with 1:1 AV conduction and LBBB aberrancy; lower panel shows 12-lead ECG and fluoroscopic image with LB area pacing and ICD upgrade. AV = atrioventricular; ECG = electrocardiogram; ICD = implantable cardioverter defibrillator; LBBB = left bundle branch block.

A 32-year-old woman presented with a history of recurrent exertional syncopal episodes. Her initial syncopal episode was after a 2-km jog in the morning and another episode the same evening while climbing up the stairs. She had no family history of sudden cardiac death. Her electrocardiogram (ECG) showed sinus rhythm, 1:1 AV conduction, and complete left bundle branch block (LBBB) (Figure 1). Twenty-four-hour Holter revealed baseline sinus rhythm with occasional ventricular premature complexes and no episodes of high-grade AV block. Her echocardiogram was normal. She was advised but declined diagnostic electrophysiology study and permanent pacemaker implantation.

Six months after the initial visit, the patient presented to the emergency room (ER) with exertional syncope. The ECG in the ER showed high-grade AV block followed by cardiac arrest. After resuscitation, the patient underwent dual-chamber permanent pacemaker implantation with left bundle branch area pacing, using the Medtronic 3830 lead in view of underlying left ventricular (LV) dysfunction during this presentation (LV ejection fraction [LVEF], –40%). The LVEF improved to normal during 2 months of follow-up.

**Figure 2:**
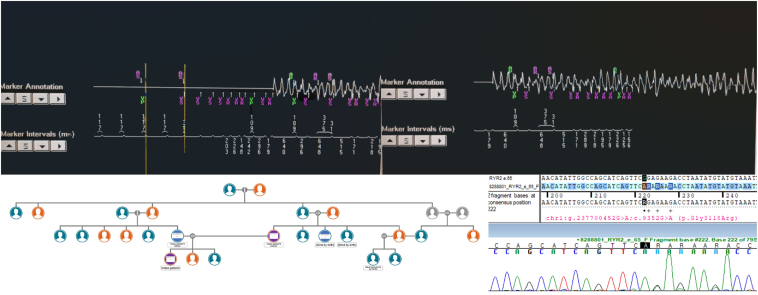
Upper panel shows 2 episodes of polymorphic ventricular tachycardia recorded on EGMs. Lower panel shows family pedigree on the left and variant detection in Sanger sequencing on the right. EGM = electrogram.

One year after the pacemaker implantation, the patient again presented with syncope in the ER after an emotionally charged dispute with her husband. The pacemaker interrogation showed normal pacemaker function; however, there were 2 runs of polymorphic ventricular tachycardia (PVT) during the syncope (Figure 2). Investigations during this time showed normal QT and normal serum potassium and magnesium levels. She was afebrile, and coronary angiogram and cardiac magnetic resonance imaging done during this time were normal.^1^ She was started on beta-blocker therapy, which was progressively titrated to the maximum tolerated dose (metoprolol tartrate, 100 mg twice per day), and the device was upgraded to a dual-chamber DF-1 implantable cardioverter defibrillator with preexisting left bundle area pacing. Positron emission tomography computed tomography done after 3 months of the implantable cardioverter defibrillator upgrade was negative for inflammatory cardiomyopathy.

A panoramic family history was again taken, and although there were no family members with history of sudden cardiac death, she was a product of a second-degree consanguineous marriage. Two maternal uncles (mother’s brothers) were blind by birth, and another maternal cousin had congenital deafness.

In view of her young age, cardiac conduction system disease, and stress-induced polymorphic VT, and absence of inflammatory cardiomyopathy and ischemia, inherited arrhythmogenic cardiomyopathies such as CPVT, SCN5A mutation, and laminopathies were strongly considered.^2^^,^^3^

On genetic testing, a pathogenic homozygous missense variant in exon 65 of the RYR2 gene (chr1: g.237700452G>A; Depth: 66x) that results in the amino acid substitution of arginine for glycine at codon 3118 (p. Gly3118Arg; ENST00000366574.7) was discovered, which is a novel mutation of the RYR2 gene mutation resulting in CPVT.

We strongly counseled all family members to undergo cascade genetic screening and were able to perform genetic testing in 4 of the index patient’s family—the mother, the father, 1 of the maternal uncles, who was blind, and the cousin who was hearing impaired. The cascade screening showed the presence of the same RYR2 mutation-Gly3118Arg (heterozygous) in 3 of the 4 tested relatives (Figure 2 lower panel, left)—the mother, the father, and the maternal uncle of the index patient. All 4 family members underwent an exercise stress test, which did not provoke any arrhythmias. This strongly suggested an autosomal recessive pattern of inheritance, with the index patient harboring a biallelic homozygous mutation caused by a chance consummation of her parents, who were carrying the causative mutation in a heterozygous form.

The index patient has had no further episodes of PVT during the last 12 months of follow-up on tablet metoprolol tartrate 100 mg twice per day. She has also undergone behavioral therapy on avoiding situations that may cause excessive adrenergic activation. Her family members have been provided with genetic counseling and have been strongly advised regarding the need to avoid consanguineous marriage.

## Discussion

Our index case provides evidence for a careful consideration of a genetic disorder in the presence of symptomatic AV conduction system disease in a young subject. CPVT caused by an RYR2 mutation characteristically shows an autosomal dominant pattern of inheritance; however, none of the immediate or extended family members had any history of syncope or sudden cardiac death. The presence of the autosomal recessive mutation in unaffected family members who are carriers also underlines the need for cascade family screening and genetic counseling.

The clinical spectrum of CPVT includes progressive AV block, SA node dysfunction, atrial fibrillation, atrial standstill, and arrhythmogenic right ventricular dysplasia features.^4^, ^5^, ^6^

AV block with an RYR2 mutation has been described by Petrungaro et al^7^ in an Italian family. The index patient, the father, was implanted with a permanent pacemaker for complete AV block at 55 years of age, and 3 of his children showed bidirectional VT and PVCs during exercise and extracardiac manifestations of speech and epilepsy. All 4 affected family members showed evidence of noncompaction. However, the index patient did not develop polymorphic VT and was on medical follow-up.

The same novel G3118R mutation has been previously described by Shauer et al^8^ in a large family from a village in Jerusalem, with multiple family members having a history of sudden cardiac death. All of the affected family members were homozygous for the same mutation, and the heterozygous individual did not manifest any symptoms. Many of the affected individuals were products of consanguineous marriage, as in our case. The case report does not elaborate on the noncardiac manifestations in the heterozygous carriers of the mutation.

Traditionally the RYR2 mutations are mostly clustered into 4 regions in the protein sequence of the N-terminal region, which forms the gating ring at the cytosolic side, the pore region, and other transmembrane-forming regions, including the C-terminal domain.^9^ They are typically inherited in an autosomal dominant manner and cause gain of function of the protein, resulting in diastolic Ca2+ leakage from the sarcoplasmic reticulum, which leads to cytosolic Ca2+ overload, driving the membranous sodium–calcium exchanger and creating delayed afterdepolarizations, triggered activity, and ventricular arrhythmias.^10^

The RyR2 (G3118R) is a novel missense mutation located far from the known cluster mutation regions, and the clinical expression is evident only in homozygotes. This may indicate that the effect of this mutation on the channel function acts in a dose-dependent manner. In heterozygous individuals, only 2 of the 4 tetramers are affected, resulting in normal channel function and no symptoms, whereas a homozygous individual presents with polymorphic VT, as evidenced in our case, probably because all 4 monomers of the RYR2 tetramer are mutated.^11^^,^^12^

The mechanism by which the RYR mutation caused conduction system disease in our patient is not known. Studies in mice have shown effects of RYR on AV node with inhibition of RYR, resulting in decreased automaticity of the AV node. The reduced RYR expression with aging also decreases background excitability or depolarization reserve of the nodal cells in AVN conduction. It is intriguing to note that our patient who presented with LBBB initially went on to develop high-grade AV block and subsequent polymorphic VT.^13^

## Conclusion

This case report underscores the importance of considering the RyR2 mutation in the workup of complete AV block and polymorphic VT in young patients, even in the absence of significant family history of SCD, especially if the patient is born out of a consanguineous marriage. The varying penetrance and expressivity of the RyR2 gene mutation may result in different clinical phenotypes.

## Disclosures

The authors have no conflicts of interest to disclose.
